# Seizures and epilepsy in patients with ischaemic stroke

**DOI:** 10.1186/s42466-021-00161-w

**Published:** 2021-12-06

**Authors:** Johann Philipp Zöllner, Friedhelm C. Schmitt, Felix Rosenow, Konstantin Kohlhase, Alexander Seiler, Adam Strzelczyk, Hermann Stefan

**Affiliations:** 1 Department of Neurology and Epilepsy Center Frankfurt Rhine-Main, Center of Neurology and Neurosurgery, Goethe-University Frankfurt, Schleusenweg 2-16, 60528 Frankfurt am Main, Germany; 2 LOEWE Center for Personalized Translational Epilepsy Research (CePTER), Goethe-University Frankfurt, Frankfurt am Main, Germany; 3 Department of Neurology, University Hospital Magdeburg, Magdeburg, Germany; 4 Department of Neurology - Biomagnetism, University Hospital Erlangen, Erlangen, Germany

**Keywords:** Cerebral ischaemia, Mortality, Antiepileptic drug, Anticonvulsants, Thrombolysis, Mechanical thrombectomy, Biomarkers, Prognosis

## Abstract

**Background:**

With the increased efficacy of stroke treatments, diagnosis and specific treatment needs of patients with post-stroke seizures (PSS) and post-stroke epilepsy have become increasingly important. PSS can complicate the diagnosis of a stroke and the treatment of stroke patients, and can worsen post-stroke morbidity. This narrative review considers current treatment guidelines, the specifics of antiseizure treatment in stroke patients as well as the state-of-the-art in clinical and imaging research of post-stroke epilepsy. Treatment of PSS needs to consider indications for antiseizure medication treatment as well as individual clinical and social factors. Furthermore, potential interactions between stroke and antiseizure treatments must be carefully considered. The relationship between acute recanalizing stroke therapy (intravenous thrombolysis and mechanical thrombectomy) and the emergence of PSS is currently the subject of an intensive discussion. In the subacute and chronic post-stroke phases, important specific interactions between necessary antiseizure and stroke treatments (anticoagulation, cardiac medication) need to be considered. Among all forms of prevention, primary prevention is currently the most intensively researched. This includes specifically the repurposing of drugs that were not originally developed for antiseizure properties, such as statins. PSS are presently the subject of extensive basic clinical research. Of specific interest are the role of post-stroke excitotoxicity and blood–brain barrier disruption for the emergence of PSS in the acute symptomatic as well as late (> 1 week after the stroke) periods. Current magnetic resonance imaging research focussing on glutamate excitotoxicity as well as diffusion-based estimation of blood–brain barrier integrity aim to elucidate the pathophysiology of seizures after stroke and the principles of epileptogenesis in structural epilepsy in general. These approaches may also reveal new imaging-based biomarkers for prediction of PSS and post-stroke epilepsy.

**Conclusion:**

PSS require the performance of individual risk assessments, accounting for the potential effectiveness and side effects of antiseizure therapy. The use of intravenous thrombolysis and mechanical thrombectomy is not associated with an increased risk of PSS. Advances in stroke imaging may reveal biomarkers for PSS.

## Background

Improvements in stroke treatment and rehabilitation have reduced stroke-associated mortality rates in the last decades. However, this improvement has been accompanied by an increased prevalence and relevance of post-stroke seizures (PSS), which manifest in approximately 5–7% of ischaemic stroke survivors each year [1, 2] and can worsen post-stroke prognosis. The PSS risk is higher (10–16%) in those who experience primary intracerebral, subarachnoid, or subdural haemorrhage [3–5]. PSS treatment options, including personalised medical approaches, are currently being investigated from a variety of perspectives [6].

Here, we discuss (1) the current indications and therapy for PSS; (2) prevention strategies and personalised therapeutic concepts; and (3) new research approaches.

## Current indications and drug treatment options

### Indications for antiseizure medication therapy in stroke

Seizures that occur within 7 days of any acute lesion event, such as stroke, traumatic brain injury, or brain surgery, are referred to as acute–symptomatic seizures (ASS) or “early” seizures, whereas all subsequent seizures are termed “late” seizures (LS) [7]. This distinction is based on pathophysiological reasoning and clinical observations. ASS are considered a correlate of the homeostatic disturbances in acute brain injury and thus do not per se equate epilepsy. LS, however, are presumed to occur spontaneously in a brain that is structurally predisposed to seizure generation. According to most studies, LS in stroke survivors are associated with a high risk (> 70%) of seizure recurrence, higher than after ASS [8]. Thus a single, unprovoked, LS results in diagnosis of post-stroke epilepsy (PSE) according to the current epilepsy definition established by the International League Against Epilepsy (ILAE) [9, 10], which set a threshold of > 60% recurrence risk within 10 years [10].

The American Heart Association (AHA)/American Stroke Association (ASA) guidelines do not recommend the primary preventive administration of anti-seizure medication (ASM) after stroke, even following haemorrhagic stroke, which has a higher risk for PSS compared with other stroke types [11–13]. In addition, ASM therapy is generally not recommended following ASS [12]. However, ASM therapy is generally recommended as a secondary prevention measure in established PSE; or in any case of status epilepticus (SE) [10]. Some borderline cases may warrant ASM therapy, which then must be individually determined: ASS following cerebral infarction with haemorrhagic transformation and the occurrence of multiple ASS within 24 hours can indicate short-term ASM treatment (over one month), which might reduce the risk of later seizures and PSE [14]. If ASS is associated with primary cerebral haemorrhage or cerebral venous sinus thrombosis with motor deficits, ASM treatment over several weeks can be considered, although insufficient evidence exists to support any general recommendations [15, 16]. The European Stroke Organization (ESO) guidelines [3] recommend the discontinuation of ASM administered after an ASS when the acute (stroke unit) phase has passed and the patient is transferred, but the authors caution that the current level of evidence regarding almost all recommendations for PSS treatment is very low. In consequence, treatment initiation for PSS should consider infarct and seizure characteristics, comorbidities, ASM adherence, medication tolerance, and interactions, among other factors.

Well-established risk factors for both ASS and LS following ischaemic stroke include stroke severity, cortical localisation, younger age, and haemorrhagic transformation [17]. An analysis of 135,117 patients with ischaemic stroke showed that ASS risk is associated with higher National Institutes of Health Stroke Scale (NIHSS) scores on admission [18]. While relatively mild strokes (NIHSS < 3) were associated with an ASS risk of 0.6%, the most severe stroke (NIHSS > 31) carried an ASS risk of 9%; with the odds of ASS increasing by 9.2% for every additional NIHSS point [18]. Non-neurological infections and a low premorbid functional level also increased the risk for ASS [18]. Risk scales can be used to aid decision-making, such as the Post-Stroke Epilepsy Risk Scale (PoSERS: Sensitivity 70%, Specificity 99.6%) or the SeLECT Score (Severity of stroke, large-artery atherosclerotic aetiology, early seizures, cortical involvement, territory of middle cerebral artery involvement; Sensitivity 18.2%, Specificity 96.7%, for cutoff at ≥ 6 points) [19–21]. If an unprovoked LS occurs, the patient should be informed of the recurrence risk, and ASM therapy should be recommended. Individual treatment recommendations should be thoroughly discussed with the patient, with consideration for current research findings [3] and personalised medical factors such as vocational and driving licence status or e.g. risk of seizure-associated falls. An exclusively non-severe seizure presentation (e.g. no focal impaired-awareness seizures, no focal-to-bilateral tonic–clonic seizures, and a low risk of injury during seizures) may not require ASM therapy. As the risk of neurological damage following post-stroke SE is 2–3 times higher compared to non-seizure patients (among 31 patients with SE, 15 patients died within 10 years, including 5 that died during an SE event), long-term therapy is necessary after post-stroke SE [22].

In the pre-hospital to emergency room phase, the differential diagnosis of acute cerebral infarction and postictal Todd’s paresis can be challenging if preceding positive motor seizure symptoms are not observed. In one study of 539 patients undergoing thrombolysis, 11 were retrospectively determined to have had Todd’s paresis rather than stroke [23]. Seizure-associated stroke mimics account for 85% of all mimics that receive acute stroke treatment, such as thrombolysis [24]. This diagnostic uncertainty can be consequential for therapy: Misdiagnosing stroke as seizure can delay time-critical recanalization therapy. The opposite risk associated with thrombolysis in patients with stroke-mimicing seizures however is probably much lower; the available studies on seizure-related stroke mimics did not report serious thrombolysis-related adverse reactions [24].

The differential diagnosis is further complicated by very-early “stroke-onset” seizures. Pre-existing neurological deficits must also be evaluated in the assessment of potential LS sequelae in those with a previous stroke.

### Choice of antiseizure medication in the treatment of post-stroke seizures

The long-held assumption that most patients with PSE can successfully be treated using ASM monotherapy [25] has recently been challenged [26]. This underscores the importance of thoughtful ASM selection in stroke patients, who tend to be older, especially regarding potential drug interactions. Overall, clinical studies have suggested that new-generation ASM are preferable to first-generation ASM for the treatment of PSE due to improved tolerability and reduced interactions with other drugs [27]. Among the newer ASM, lamotrigine (LTG), levetiracetam (LEV), and lacosamide (LCM) have demonstrated relatively high tolerability and unproblematic interaction profiles in PSE treatment.

LTG shows moderate antiseizure efficacy, is well tolerated, is typically mood-stabilising, has a low interaction potential, and is relatively convenient apart from the necessity of slow dose increases (once-daily administration is possible). Interestingly, LTG is better tolerated in patients with PSS than carbamazepine (CBZ), another liver-metabolized ASM [28]. Recent in-vitro data have demonstrated that the sodium channel blocker LTG acts as class IB antiarrhythmic agent at therapeutic serum levels. Possible proarrhythmogenic properties prompted addition of a warning to the label by the United States Food and Drug Administration (FDA) [29]. In the absence of clinical data, the ILAE pragmatically recommends to obtain an electrocardiogram (ECG) before start of LTG in those with known cardiac disease, cardiovascular risk factors and those above 60 years of age to rule out relevant cardiac conduction abnormalities [29]. Most stroke patients fall into these categories, however, a thorough cardiologic work-up including routine and in some cases long-term ECG is part of standard-of-care in stroke patients, which increases the likelihood that pre-existing cardiac conditions have already been identified. An ECG should be repeated in those with cardiac disease at target dose.

LEV is associated with high antiseizure efficacy, a low interaction potential, and can be administered in an intravenous formulation for the rapid achievement of effective serum concentrations and use in patients with impaired swallowing. Adverse reactions following LEV administration occur in fewer than 10% of patients (irritability and mood swings). In a prospective open-label study on LEV treatment of late post-stroke seizures [30], 77.1% of patients remained seizure-free for one year. Four patients (11.4%) discontinued LEV due to intolerable side effects (tiredness in one patient and aggressive behaviour in 3 patients) [30, 31]. In another prospective randomized open-label study, no significant difference in effectiveness was observed between LEV and controlled-release CBZ, but LEV was better tolerated [32].

LCM is generally well-tolerated and effective in patients with epilepsy of cerebrovascular etiology [33] and intravenously administered LCM showed high efficacy and tolerability in non-convulsive SE (NCSE) following stroke in patients older than 70 years [34].

Gabapentin (GBP) likely has lower antiseizure efficacy and requires multiple daily doses but has a low interaction potential. Of note, GBP carries the risk of dizziness, vertigo and altered mental status in elderly patients, especially with higher daily doses [35].

Head-to-head comparisons from randomised-controlled trials are not available specifically for efficacy in PSE. However, the STEP-ONE trial compared LEV, LTG, and controlled-release CBZ as initial monotherapy for focal epilepsy in older individuals using a randomised setting [36]. The trial showed that the one-year retention of LEV was higher than that of CBZ due to better tolerability, whereas LTG retention was intermediate but did not differ significantly from either comparator [36]. In the recently published SANAD II study, LTG showed a better 12-month seizure remission rate than both LEV and zonisamide following the initial monotherapy of focal epilepsies [37].

Eslicarbazepine (ESL), LCM, oxcarbazepine (OXC), perampanel (PER), and zonisamide are currently underinvestigated in PSE [38–40]. An exploratory pilot study showed that LCM was relatively effective in patients with epilepsy with cerebrovascular aetiologies, with high tolerability, assuming that appropriate care is taken regarding contraindications (most importantly, certain cardiac conduction disorders). Data for monotherapies suggested both a better antiseizure efficacy and favourable pharmacokinetic profile (i.e. fewer interactions and less negative influence on lipid concentrations) for LCM in comparison to CBZ [33]. Publications on the clinical effectiveness and tolerability of ASM for PSE treatment are summarised in Table 1.

**Table 1:** Publications reporting the clinical efficacy and tolerability of antiseizure medication for treatment of post-stroke epilepsy

Author	Study design	Participants (n)	Age (years)	Medication (mg)	Period	Seizure recurrence	Tolerability	Limitations
Alvarez-Sabin et al. [38]	Prospective Observational	48 ischaemic 23 haemorrhagic	63.9	GBP 900–1800 mg	30 months	18%	Adverse events 38%; discontinued 3%	SN, NR, NP
Gilad et al. [28]	Prospective Randomised	64 ischaemic	LTG 67.2 CBZ 67.7	LTG 25–200 mg CBZ 100–600 mg	12 months	LTG 28% CBZ 56%	Discontinued LTG 3%, CBZ 31%	SN, NP, NDB
Kutlu et al. [116]	Prospective Observational	34 ischaemic	69.8	LEV 1000–2000 mg	17.7 months	18%	Discontinued 21%; stopped 3%	SN, NR, NP
Belcastro et al. [30]	Prospective Observational	35 ischaemic	71.9	LEV 1000–2000 mg	18 months	9%	Discontinued 11%	SN, NR, NP
Consoli et al. [32]	Prospective Randomised	79 ischaemic 27 haemorrhagic	LEV 74.1 CBZ 54	LEV 52 CBZ 54	13,5 months	LEV 6% CBZ 15%	Discontinued LEV 33%, CBZ 39%	SN, NP, NDB
Tanaka and Ihara [117]	Retrospective Observational	69 ischaemic 43 haemorrhagic	72.3	23 VPA, 22 PHT 15 CBZ	12 months	VPA 48% PHT 18% CBZ 13%	–	SN, mono- and polytherapy
Huang et al. [118]	Retrospective Observational	1729 ischaemic 1893 haemorrhagic	60.3	PHT 2507 VPA 712 CBZ 157 Newer ASM 246	100 person - months	PHT 1.05% (ER visits) VPA 0.7% CBZ 0.4% Newer ASM 0.38%	–	Seizure in first 3 months excluded
Sales et al. [39]	Retrospective Observational	76 PSE 1590 EPI*	PSE 63 EPI 61.4	ESL/PSE 887 ESL/EPI 983	12 months	51.4% 68.3%	Adverse events 36% versus 35.8%	Multicentric, differences between cohorts

Publications reporting the clinical effectiveness and tolerance for anticonvulsants used for the treatment of PSE, modified after Tanaka and Ihara [117]. Abbreviations: ASM: anti-seizure medication, CBZ: carbamazepine, EPI: epilepsy not associated with stroke (* with differences in age, length of preceding epilepsy treatment), ER: emergency room, ESL: eslicarbazepine, GBP: gabapentin, LEV: levetiracetam, LTG: lamotrigine, NDB: not double-blind, NP: no placebo, NR: non-randomised, PHT: phenytoin, PSE: post-stroke epilepsy, SN: small number of patients; VPA: valproate

CBZ, phenytoin (PHT), and valproate (VPA) are not first-line ASM among older patients due to their lower tolerability and considerable interaction profiles. Of particular concern, a marked reduction was observed for direct-acting oral anticoagulant (DOAC) serum concentrations following CBZ and PHT administration, and CBZ and ESL may inhibit simvastatin [41–44]. CBZ, OXC, and ESL can also cause hyponatraemia, particularly in older people [45, 46].

**Fig. 1:**
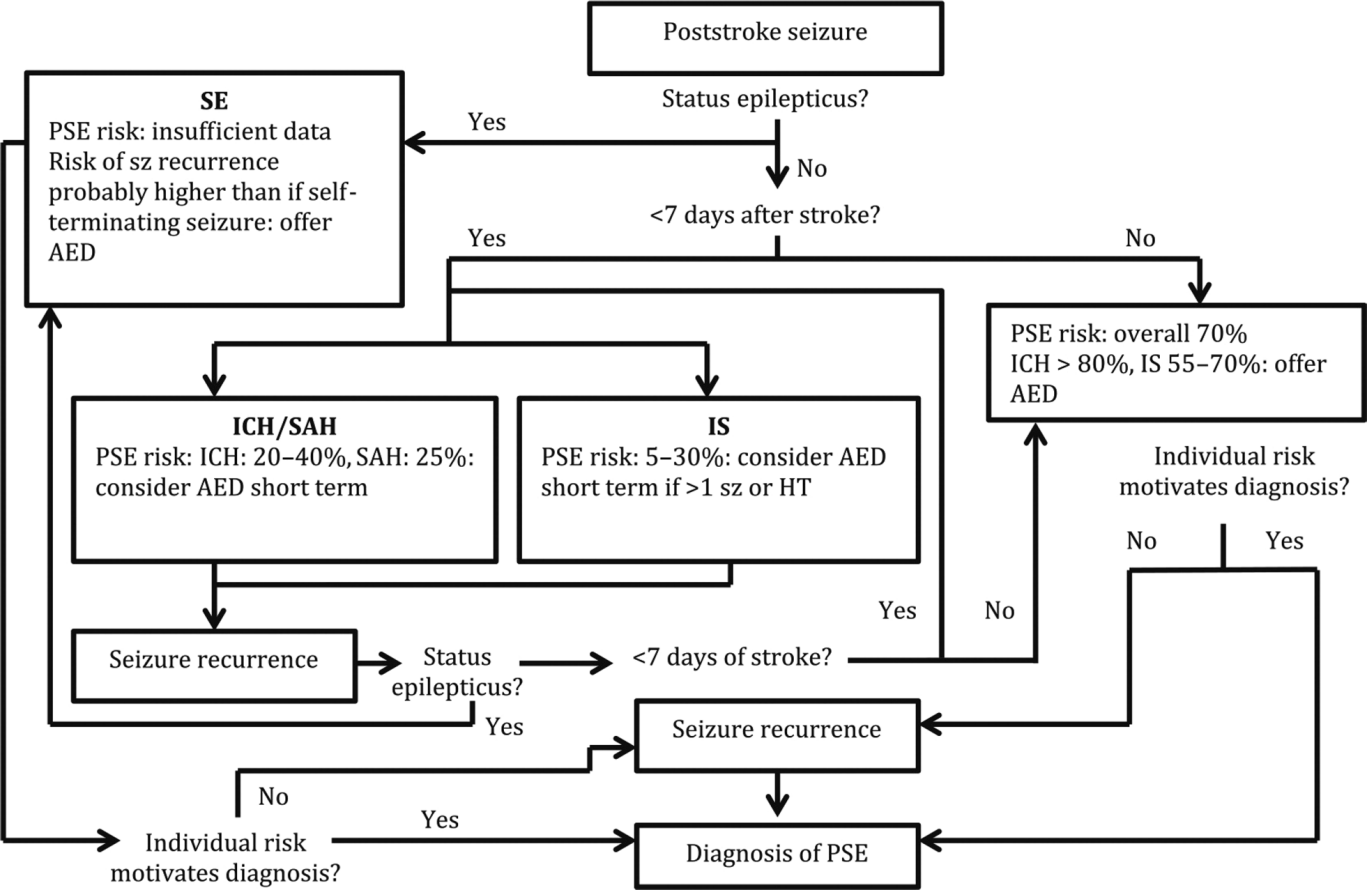
Risk-based therapeutic strategy for post-stroke seizures (PSS). AED: anti-epileptic drug, ICH: intracerebral haemorrhage, IS: ischaemic stroke, HT: haemorrhagic transformation, PSE: post-stroke epilepsy, SAH: subarachnoid haemorrhage, SE: status epilepticus, Sz: seizure. Figure adapted from Zelano J. 47, Ther Adv Neurol Disord 9(5) pp 424–435, copyright © 2016 The Author. Reprinted by permission of SAGE Publications

A risk-based therapeutic strategy for ASS or LS is schematically reproduced in Fig. 1, based on the strategy described by Zelano [47].

## Interrelation of therapies for stroke and post-stroke epilepsy

### Thrombolysis, mechanical thrombectomy and post-stroke seizures

Does thrombolysis increase the risk of PSS? Thrombolysis has been suggested as a potential PSS risk factor since the thrombolytic agent recombinant tissue plasminogen activator (rt-PA) can have neurotoxic effects on the infarcted brain [48, 49]. Successful reperfusion itself is a possible ASS-generating mechanism.

Initially, case reports suggested that ASS during recanalisation resulted in neurological improvements [50]; since then, further results have supported the notion of reperfusion as seizure generator [51]. Brigo et al. [52] showed that intravenous (i.v.) rt-PA administration (odds ratio [OR]: 2.26) independently increases the risk of ASS. Alvarez et al. [48] described frequent ASS following thrombolysis, associated with a worse prognosis.

Thrombolysis emerged as an independent risk factor also for LS in a population-based, retrospective cohort study [53]. Naylor et al. [54] observed an increase in LS occurrence as late as 24 months after thrombolysis. Analysis of various treatment groups (i.v. rt-PA, intra-arterial thrombolysis [IAT], or both) showed that all reperfusion therapies were associated with a similar increase in seizure frequency, compared with conservative stroke treatments (i.v. rt-PA corrected OR [cOR]: 3.7, 95% confidence interval [CI]: 1.8–7.4, *p* < 0.001; IAT cOR: 5.5, 95% CI 2.1–14.3, *p* < 0.001; IAT + i.v. rt-PA cOR: 3.4, 95% CI 0.98–11.8, *p* = 0.05) [54]. Castro-Apolo et al. found a similar effect on prognosis in those treated with i.v. rt-PA who had LS [55]. Brondani et al. also showed that PSS are an independent risk factor for worse prognosis following thrombolysis for stroke [56]. In this study [56], neurological deficits following thrombolysis and haemorrhagic transformation were independent risk factors for PSS.

Despite successful reperfusion and potential neurotoxicity being mechanisms for PSS generation, more recent, larger studies have uniformly demonstrated that recanalization overall is not associated with an increased risk for either ASS or LS. In a recent meta-analysis, seizure frequency (ASS 3.14%, LS 6.7%) after treatment with i.v. rt-PA, mechanical thrombectomy (MT), or both, was similar to the incidence in large, unselected patient databases, and the pooled analysis did not find significantly increased odds of PSS in those with rt-PA or MT [57, 58]. A meta-analysis of over 30 studies performed by Gasparini [59] indicated a PSE prevalence of 7%. Cortical lesions (OR: 3.58, 95% CI 2.35–5.46, *p* < 0.001), haemorrhagic components (OR: 2.47, 95% CI 1.68–3.64, *p* < 0.001), ASS (OR: 4.88, 95% CI 3.08–7.72, *p* < 0.001), and young age (difference in means with/without PSE: 2.97 years, 95% CI 0.78–5.16, *p* = 0.008) were more often associated with PSE. Therapy with rt-PA was again not identified as an independent risk factor for PSS. Consistently, Keller et al. [60] could not determine an influence for i.v. rt-PA on PSS frequency. A newer analysis by Zöllner et al. of 13,356 patients who were treated with thrombolysis for ischaemic stroke did not show a higher incidence of early seizures compared to not thrombolysed patients (1.5% vs. 1.8%, *p* = 0.07) [18]. In addition, among 1,013 patients who were treated with thrombolysis and MT, early seizure incidence was not higher than with thrombolysis alone (both 1.7%, *p* = 1) [61]. The finding that MT does not increase PSS risk was supported by a recent prospective study on 344 patients with ischaemic stroke [51]. De Reuck et al. [62] and Nesselroth et al. [63] even reported that thrombolysis partially prevented PSE, which was attributed to tissue salvage in thrombolysed patients. Kim et al. [64] suggested that rt-PA has a protective effect on brain tissue. In an further study [65] comparing 177 patients treated with i.v. rt-PA to 158 who were not specifically treated, no increase in PSS incidence was attributed to rt-PA [65]. An overview of the studies examining the risk factors for seizure development following thrombolysis can be found in Table 2.

**Table 2:** Studies reporting risk factors for post-stroke seizures after thrombolysis or mechanical thrombectomy

Author	Research subject	Patients	Results	Risk factors (independent)	Method characteristics
Alemany et al. [51]	mechanical thrombectomy; ASS and PSE	344 patients with ischaemic stroke and NIHSS > 8 treated with thrombectomy	21 (6.1%) presented ASS, The accumulated PSE incidence at 5 years was 8.93%; rt-PA not an independent risk factor for ASS	for ASS: degree of reperfusion: OR 2.02	1 centre, retrospective, observation period > / = 5 years
Alvarez et al. [48]	rt-PA and PSS prognosis	28 of 2,327 patients had PSS (1.2%)	Worse outcome in rt-PA and PSS versus rt-PA without PSS	for ASS: Cortical involvement: OR 7.5, rt-PA: OR 4.6	PSS < 7 days, mostly < 72 h, 1 centre, 3-month period
Bentes et al. [49]	rt-PA versus no thrombolysis	101 patients rt-PA; 50 no rt-PA	Seizure symptoms during rt-PA infusion 5% (*p* = 0.726 and *p* = 0.4); no difference in seizure frequency with/without rt-PA	–	Observation period 1 year, 1 centre
Brigo et al. [52]	rt-PA effect on seizure development < 7 days	79 patients	rt-PA OR 2.26	for ASS: Cortical localization: OR 2.49; i.v. rt-PA: OR: 2.26	1 centre, period: weeks
Brondani et al. [56]	rt-PA, PSE influence on prognosis	153 patients, rt-PA 13 patients	7% PSS, 9% PSE	for PSE: hemorrhagic transformation: OR = 3.55, mRS > / = 2 at 3 months after stroke: OR: 5.82	1 centre, observation period at least 2 years
Castro-Apolo et al. [55]	rt-PA versus no thrombolysis	42 patients with seizures, 62 without	33 early seizures, late seizures in 66.7%; no association with rt-PA (*p* = 0.25)	PSE worsens outcome	1 centre, mean observation period 20 months
De Reuck et al. [62]	rt-PA versus anticoagulant	38 patients rt-PA 269 patients OAC 769 patients antithrombotic	ASS increase as a correlate of reperfusion, partial reduction of late seizures	ASS associated with stroke severity	Cardiogenic or atherothrombotic ischaemic stroke only, 1 centre
Gasparini et al. [59]	rt-PA versus mechanical thrombectomy	26,055 patients (meta-analysis)	1800 (7%) PSE	for PSE: Cortical lesions: OR 3.58, hemorrhagic component: OR: 2.47, ASS: OR: 4.88, younger age at stroke onset (difference in means: 2.97 years)	Multicentre meta-analysis Heterogeneous time periods
Keller et al. [60]	rt-PA versus no thrombolysis; PSE	302 patients	PSE incidence: 20.6% rt-PA versus 10.7% no rt-PA; no effect of rt-PA after adjustment for other variables	for PSE: low Barthel Index at discharge; hemianopia; infection acquired during the hospital stay; involvement of the temporal lobe; involvement of the perirolandic cortex	1 centre, 42-month period (max. 80 months)
Lekoubou et al. [57]	rt-PA versus mechanical thrombectomy; PSS	13,753 patients (meta-analysis)	529 PSS with rt-PA (6.1%), PSE 6.7%, ASS 3.14%	Pooled OR: rt-RA und PSS 1.24 (not significant), no difference PSE regarding rt-PA or mechanical thrombectomy	multicentre meta-analysis
Naylor [54]	rt-PA versus IAT versus IAT + rt-PA (PSS)	363patients rt-PA: PSE 5.8% 1375 patients, stroke unit only	PSS: IAT 12.9% rt-PA + IAT 4.5% 2% PSE	for PSE rt-PA: OR 3.7 IAT: OR 5.5 rt-PA + IAT: OR 3.4	multicentric, 2-year period
Nesselroth et al. [63]	rt-PA versus antiaggregation	rt-PA 141 patients rt-PA + antiaggregation 141 patients antiaggregation only 95 patients	PSS: rt-PA 8.1% antiaggregation 12.6% rt-PA + antiaggregation 5.8%	rt-PA reduces PSS risk by 6%	1 centre, 1-year period
Tan et al. [65]	rt-PA versus no thrombolysis	177 patients rt-PA 158 patients no rt-PA	PSE: 8.25% rt-PA, no rt-PA 6.5%; no significant effect of rt-PA on PSE risk	PSE worsens functional prognosis	2-year period with phone interview, 1 centre
Polymeris et al. [119]	presence of ASS	10,074 patients rt-PA	1.5% ASS	seizures at onset not an independent predictor of outcome	Pooled data from 9 centres rt-PA with or without subsequent endovascular therapy Multicentre Various time periods
Zöllner et al. [61]	rt-PA versus mechanical thrombectomy; ASS	13,356 patients with rt-PA and 1013 patients with rt-PA and mechanical thrombectomy	ASS: 1.5% (n = 199) with rt-PA versus 1.8% (n = 237) in controls without rt-PA 1.7% with rt-PA and mechanical thrombectomy versus 1.7% (each n = 17) in controls with rt-PA only	No difference in frequency of ASS between patients with rt-PA versus no recanalisation (historical cohort) or rt-PA and mechanical thrombectomy versus rt-PA only	Study matched for age, NIHSS and premorbid function level with population-based register data

Studies of risk factors for seizure development following thrombolysis. aOR: adjusted odds ratio, ASS: acute symptomatic seizures, IAT, intra-arterial therapy, NIHSS: National Institutes of Health Stroke Scale, OAC: oral anticoagulant, OR: odds ratio, PSE: post-stroke epilepsy, PSS: post-stroke epileptic seizure, rt-PA: recombinant plasminogen activator

In conclusion, studies of seizure development following recanalisation, thus far, have been somewhat contradictory. The largest studies and meta-analyses however indicate that neither i.v. rt-PA nor (with less certainty) MT are independent risk factors for ASS or LS when controlling for other known risk factors, such as stroke severity, cortical location and haemorrhagic transformation [57, 59, 61]. The inconsistency of past results can be ascribed to variation in investigation methods and included clinical variables, and small case numbers in single-centre studies. As an inherent methodological limitation of meta-analyses, important clinical variables may not be available at the patient level, precluding the correction for confounders and the overestimation of the effects of recanalisation on PSS risk [58, 66]. The volume and (cortical) location of successfully reperfused parenchyma as risk factor for ASS generation [51] remains understudied and deems further investigation.

### Effects of ASM on coagulation and the cardiovascular system

The European Heart Rhythm Association (EHRA) published recommendations in 2018 for the use of non-vitamin-K anticoagulants (DOAC) in patients with atrial fibrillation [67]. These suggested that the combination of LEV and DOAC might be problematic, based on considerations of P-glycoprotein (P-gp) function in animal models. Von Oertzen et al. [68] objected to this suggestion, as no clinical evidence of a LEV–DOAC interaction has been reported, and the increased risk of mortality associated with PSE following stroke should be a more serious consideration. Further pharmacogenetic studies have supported this lack-of-interaction [69, 70]. By contrast, enzyme-inducing ASM, such as CBZ, PHT, phenobarbital, and primidone, can interact significantly with common post-stroke drugs, including anticoagulants, antihypertensives, and statins, with potentially severe risks for stroke patients. The differences in interaction potential between edoxaban, dabigatran, apixaban, and rivaroxaban are summarised by Steffel et al. [67]; of note, certain anti-arrhythmic drugs can exhibit proconvulsant side effects [71].

### Primary, secondary, and tertiary prevention of epileptic seizures following stroke

Epileptic seizures following stroke can lead to the worsening of brain damage, as demonstrated by the results of a study employing diffusion-weighted magnetic resonance imaging (MRI) [72], and the determination of possible preventive measures is of great importance. Primary prevention involves the immediate prevention of seizures following stroke. Secondary prevention refers to the prevention of further seizures following an initial PSS. Tertiary prevention includes seizure recurrence prophylaxis to facilitate medicorehabilitative treatment and modify epileptogenicity after stroke.

#### Tertiary prevention

Approaches to tertiary prevention comprise individually optimised rehabilitation and antiseizure therapy. In addition to the indication, selection, and dosing of ASM, potential long-term consequences should be considered. Patients with PSE commonly present with several cardiovascular risk factors, requiring the avoidance of ASM that adversely affect biochemical markers of vascular disease, such as total cholesterol, lipoproteins, C-reactive protein (CRP), and total homocysteine, which may eliminate CBZ, PHT, phenobarbital, and primidone as options according to Mintzer et al. [73]. Chuang et al. [74] reported a significant increase in the intima-media thickness of the common carotid artery (CCA–IMT) with the long-term (> 2 years) use of older-generation ASM (CBZ, PHT, and VPA), correlating with use duration. The use of enzyme-inducers, such as CBZ or PHT, was associated with adverse changes in cholesterol, folic acid metabolism, and increased CRP. Patients also showed higher uric acid and total homocysteine levels and higher oxidation markers, such as thiobarbituric acid reactive substances (by-products of lipid re-oxidation). No significant changes in these markers or in the CCA–IMT were observed with LTG monotherapy. However, the average duration of LTG therapy in this study was shorter (5.5 ± 3.1 years) than of the other medications (CBZ: 13.4 years, PHT: 10.7 years, and VPA: 8.7 years). When comparing CBZ, PHT, and VPA, a particularly strong association with increased high-density lipoprotein cholesterol was observed for CBZ and PHT [75]. This suggests a possible link to the observation that statin therapy can reduce the risk of PSS recurrence, independent of the secondary preventative effect of statins on strokes themselves [17].

#### Secondary prevention

The indications for secondary prevention after a single PSS are discussed above. Of note, long-held assumptions that seizure control is easier achievable in PSE compared with focal epilepsy in general [25] have recently been challenged [26].

#### Primary prevention

It is worth noting that current guidelines do not recommend primary preventative antiseizure treatment of patients with ischaemic stroke [3, 12]. While primary prevention of PSS and PSE is the subject of extensive research efforts, trials that are adequately powered to guide treatment are still lacking [3]. In practice, primary prevention refers to the exploitation of the additional antiepileptogenic effects of a drug rather than its intended pharmacologic properties. However, heterogeneity among lesions, dosages, and the initiation and duration of treatment has made definitive assertions regarding the antiepileptogenic properties of drugs and their potential clinical relevance difficult to achieve, resulting in a lack of translational studies. Klein et al. [76] provided a comprehensive overview of animal studies and clinical data on antiepileptogenic effects of various drugs [76]. Here, we discuss several possibly relevant drugs: Potential antiepileptogenic properties have been ascribed to LEV and GBP [77–79]. Interestingly, the particular nature of existing post-stroke data has led to the discovery of potential antiepileptogenic effects for several drugs apart from their intended use. For example, the antihypertensive drugs losartan and telmisartan, both angiotensin-type 1 receptor (AT1) antagonists, have been proposed to have antiepileptogenic effects based on studies examining the role of the blood–brain barrier (BBB) in epileptogenesis [76]. BBB disruption allows albumin to enter the brain, where it binds transforming growth factor-beta (TGFβ) receptors on astrocytes, triggering the release of proinflammatory cytokines, driving epileptogenesis [80, 81]. AT1 antagonists can inhibit TGFβ activation and prevent epileptogenesis by blocking Smad 2/3 phosphorylation following BBB disruption or the direct exposure of the cerebral cortex to albumin [82]. The diuretics thiazide and furosemide have been shown in animal and clinical studies to reduce seizure frequency [83]. Statins reduce the risk of epilepsy-related hospitalisation in patients with cardiovascular disease, whereas several ASM have demonstrated no such effect [84]. In a study by Guo et al., statins were shown to be associated with a reduced risk of PSE [17], a finding that was confirmed by Li et al. [85], who noted that statin use reduced the risk of both ASS and PSE (both *p* = 0.009). The risk reduction was even stronger with high-dose statin treatment (ASS *p* = 0.003, PSE *p* = 0.006) and improved with longer-term versus short-term treatment (*p* = 0.015), possibly due to the anti-inflammatory properties of statins. A systematic review of statin use for the primary prevention of PSS and PSE by Nucera et al. [86] reported that one study showed reduced ASS risk and three studies showed reduced PSE risk with statin use following haemorrhagic cerebral infarction.

Other drugs, such as rapamycin, have antiepileptogenic mechanisms but have not yet been investigated in PSE [87]. The glutamate receptor antagonist PER was able to prevent the overactivity of glutamate receptors and block ischaemic pathological long-term potentiation. The neuroprotective antiseizure effects of PER could be observed at very low doses. Zonisamide showed similar neuroprotective effects to PER [88].

## Current research approaches

### Imaging of post-stroke glutamate-mediated excitotoxicity

The importance of thrombolysis for the primary prevention of PSS requires further clarification. Persistent neurological clinical deficits following thrombolysis (rather than the initially presenting deficits) are associated with a worse outcome and are independently associated with PSS or PSE [56]. Important questions remain to be investigated: Which mechanism is more relevant for epileptogenicity: the preservation of brain tissue by reperfusion or potential haemorrhagic transformation? Why does the outcome become less favourable when PSS occurs [18, 51]? Why do those with LS and a certain percentage of patients with ASS develop PSE? Animal models, such as the photothrombotic stroke model, provide a perspective for better understanding the mechanisms underlying epileptogenesis following cerebral infarction [89]. Through experimental models, changes can be analysed at the molecular and cellular levels and at the network level following stroke. Biomarkers can contribute to understanding the pathophysiology associated with PSS and improve risk assessment. Glutamate plays a significant role in epileptogenesis via post-stroke excitotoxicity, and the measurement of post-stroke glutamate concentrations may be useful [90]. The 7-Tesla chemical exchange saturation transfer (CEST) MRI method can be used to non-invasively measure glutamate (*Glu*CEST) by indirectly measuring metabolite concentrations (e.g. glutamate) based on the energy transfer between hydrogen protons bound to the metabolite of interest and surrounding free water protons. A magnetically saturated, energetically excited state is induced in the metabolite-bound protons using a radiofrequency pulse shifted to the metabolite protons’ resonance frequency to saturate the metabolite signal and reduce the MR signal. The spontaneous transfer of saturated protons to surrounding water results in a reduced MRI water signal proportional to the protons saturation quantity. The difference in water signal can be used as indirect evidence of metabolite concentration, such as glutamate. Repeated excitations increase the detectability of the molecule. As a proof of concept, an animal study showed a 100% increase in the *Glu*CEST signal following middle cerebral artery occlusion [91].

**Fig. 2:**
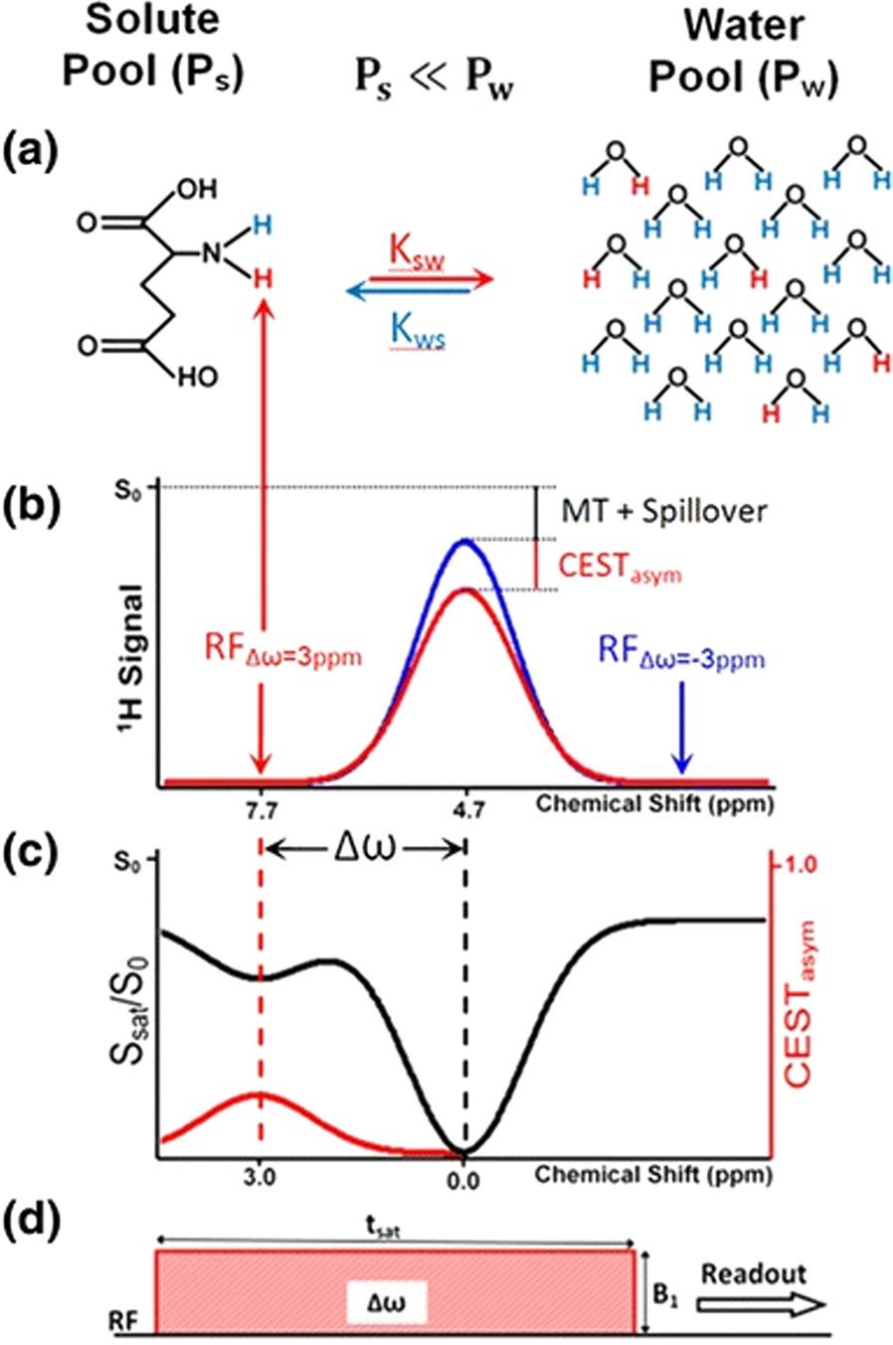
Transfer of hydrogen protons and the resulting difference in water signal as an indicator of glutamate concentration using the *Glu*CEST method [92]. Figure reproduced from Kogan et al. [92], Curr Radiol Rep 1 pp 102–114. Reprinted by permission from Springer Nature: Copyright © Springer 2013

Figure 2 shows the transfer of hydrogen protons and the resulting difference in the water signal as an indicator of glutamate concentration.

**Fig. 3:**
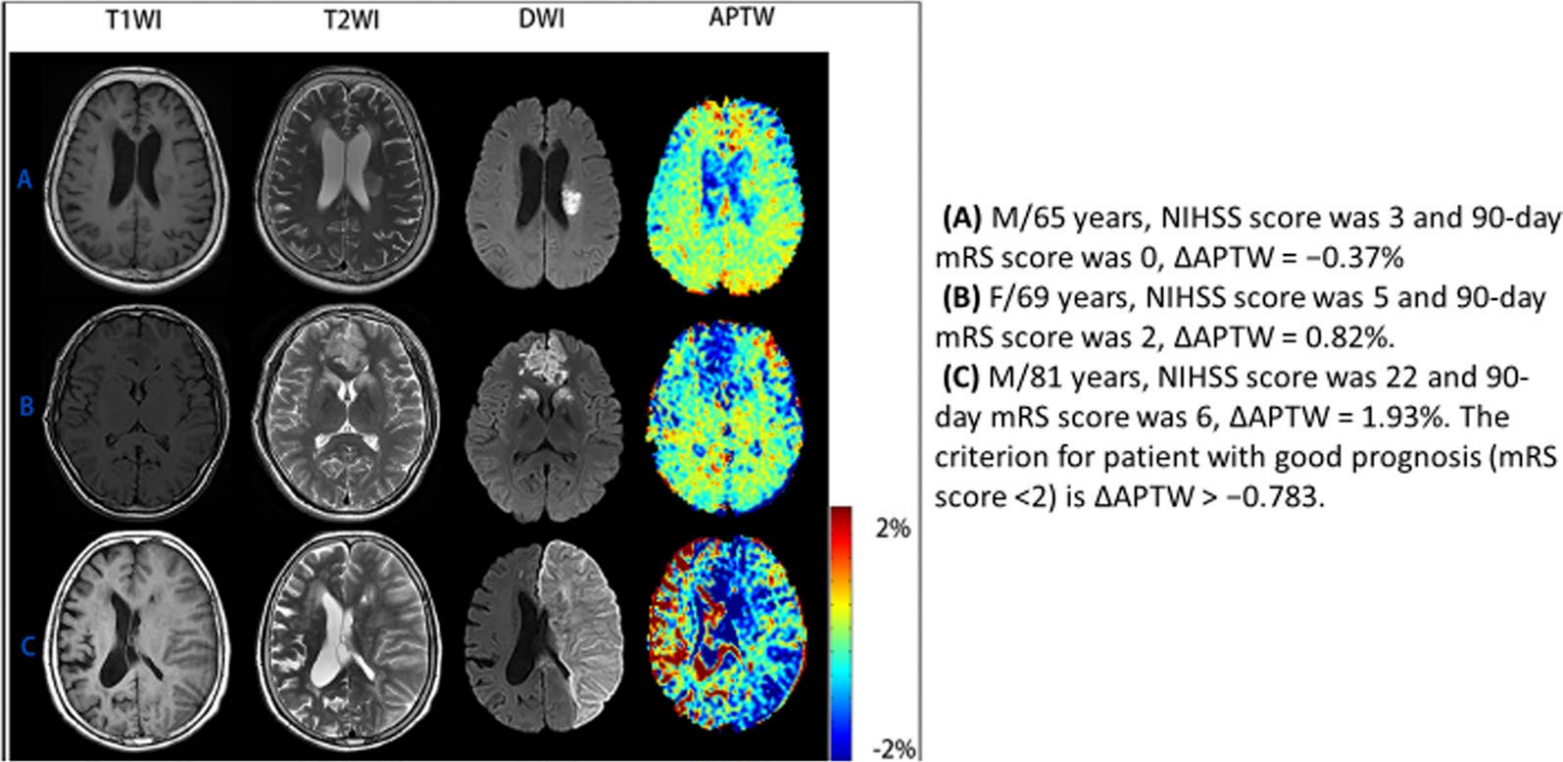
Conventional structural MR images (T1- and T2-weighted imaging [T1WI/T2WI]) in the first and second column, diffusion-weighted image (DWI) in the third column, and amide proton transfer weighted imaging (APTW) in the fourth column from the left. Each row corresponds to images from an individual patient with acute ischaemic infarct and differing clinical reports [93]. Figure reproduced according to the Creative Commons Attribution (CC BY) license from: Lin et al. [93]. Copyright © 2018 Lin, Zhuang, Shen, Xiao, Chen, Shen, Zong and Wu

Another CEST analysis method uses a pH-weighted process to measure amide proton transfer signal intensity. In 55 patients with acute ischaemic infarct, the change in amide proton transfer signal intensity showed a good correlation (*p* < 0.001) with the NIHSS value and the 90-day modified Rankin scale (mRS) value (*p* < 0.001), which may offer a method for estimating stroke severity and long-term prognosis (Fig. 3) [93].

To date, no unequivocal clinical evidence has been reported regarding the effectiveness of antiepileptic primary PSS prevention using medication. However, non-selective competitive α-amino-3-hydroxy-5-methyl-4-isoxazolepropionic acid (AMPA) receptor antagonists with glutamate-modulating effects, such as PER, represent possible antiepileptogenic and neuroprotective treatment options. *Glu*CEST analyses may deliver further insights regarding the increased risk of PSS associated with increased glutamate signalling, whether antiglutamatergic agents can be used for primary PSS prevention purposes, and how the antiepileptogenic/neuroprotective effects of this approach compare against those associated with other drugs.

The relevance of biomarkers for risk assessment has been demonstrated through other research approaches. The evaluation of intracerebral bleeding using the microRNA (miRNA) regulatory network as a potential biomarker for PSE confirmed that two miRNAs (4317 and 4315) are differentially expressed in PSE. The miRNA mi4317 regulates SCLC38A1, a glutamine-glutamate transporter [94]. The search for blood biomarkers that might be predictive for PSE confirmed known clinical risk factors, such as an NIHSS score of ≥ 8 (*p* < 0.001) and ASS occurrence (*p* < 0.001), and identified significant and independently associated serological markers, including an endostatin concentration > 1.23 ng/ml (*p* = 0.046) and low concentrations of S100B and heat shock proteins (Hsp70 < 2.496 ng/ml, *p* = 0.006). The risk of PSE associated with combined supra-threshold values of these two biomarkers was 17%. A combination of clinical and blood biomarkers further increases predicted risk [95]. These results complement prior findings exploring reduced tumour necrosis factor receptor 1 (TNF-R1) levels and increased levels of neural cell adhesion molecule (NCAM) as risk factors for post-stroke ASS [96]. NCAM is expressed on the pre- and post-synaptic membrane, where it mediates cell contacts between neurons and participates in the production of neurites and learning processes. NCAM is a danger-associated molecular pattern (DAMP) protein, which refers to a family of biomolecules that initiate inflammatory processes and are released during the neuroinflammatory phase. Another potential PSE biomarker is the polymorphism of acetaldehyde dehydrogenase 2 mitochondrial enzyme–rs671 (ALDH2–rs671) [97]. If confirmed, biomarker-based or combined clinical-biomarker based risk estimates could increase our ability to individually predict the occurrence of ASS and LS and thus contribute to the primary and secondary prevention of PSS.

### Blood–brain barrier dysfunction and post-stroke seizures

The critical contribution of BBB dysfunction to the development of epileptic seizures and epilepsy has been widely acknowledged, although the mechanisms underlying epileptogenesis in pathologies associated with primary or secondary BBB damage are not completely understood [98, 99]. Among the mechanisms associated with BBB damage that might promote and maintain ictal activity at the cellular level, specific attention has been paid to the early astrocytic response to the extravasation of serum proteins, resulting in the activation of the innate immune system and altered potassium and glutamate homeostasis [99]. As a consequence of these changes, neuronal excitability is enhanced and potentially propagated via network connections [99]. Consistent with the link between BBB disruptions and epileptogenesis, DAMP protein levels, which serve as indicators of BBB dysfunction, are increased in stroke patients who later developed PSS [98, 100].

**Fig. 4:**
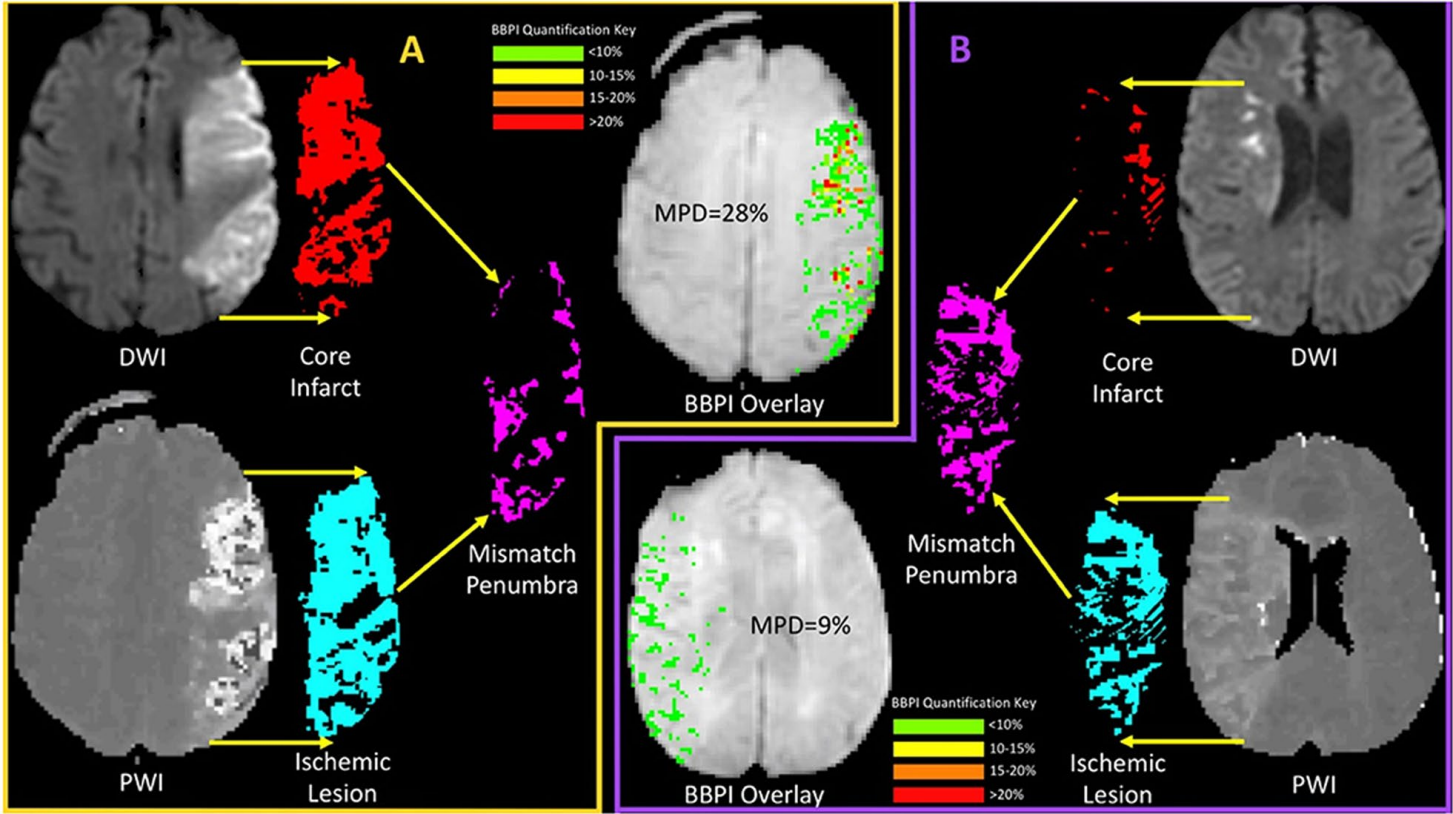
Examples of blood–brain barrier permeability images using dynamic susceptibility contrast-enhanced perfusion-weighted imaging (DSC-PWI) showing the relative increase of BBB permeability in the area of ischemia compared to the corresponding area of the unaffected hemisphere (upper right image, respectively lower left image in (**a, b**)) [108]. Figure reproduced according to the Creative Commons Attribution (CC BY) license from: Heidari et al. (2020). Copyright © 2020 Heidari, Blayney, Butler, Hitomi, Luby and Leigh

Using MRI techniques, the occurrence of BBB disruption in acute ischaemic stroke has been increasingly investigated, particularly with regard to associated demographic and clinical factors, the impact of reperfusion therapies, and the prognostic relevance of BBB dysfunction for predicting haemorrhagic transformation and clinical outcomes after stroke [101]. For the assessment of BBB disruption in the setting of acute stroke, perfusion-weighted imaging (PWI) is a particularly useful technique that can easily be incorporated into a standardised stroke imaging protocol and has been widely used [102–105]. The PWI approach that has traditionally been applied to the investigation of pathological changes in BBB permeability is dynamic contrast-enhanced imaging (DCE, often referred to as permeability imaging) and considers differences in the pre- and post-contrast T1-weighted images [102]. However, bolus-tracking dynamic susceptibility contrast imaging (DSC), which is included in standardised clinical stroke imaging protocols for the assessment of tissue-at-risk and therapeutic decision-making at many stroke centres [106], can also be employed to investigate BBB damage in acute stroke [105, 107], as the echoplanar imaging (EPI) sequences employed for DSC feature a mild T1-weighting in addition to being predominantly T2*-weighted [103, 105]. However, patients exhibiting a cerebral perfusion deficit, such as in acute stroke, require an arrival time correction for DSC-based permeability imaging to control for altered blood flow effects before calculating the permeability signal [103, 105]. Both the DCE-PWI and delay-corrected DSC-PWI techniques to assess BBB permeability have successfully been applied successfully to investigate pre- and post-treatment BBB leakage in previous studies, which demonstrated associations between BBB disruption and haemorrhagic transformation, parenchymal haemorrhage, and unfavourable clinical outcomes [102, 104, 105, 107, 108] (Fig. 4). Furthermore, a high magnitude of BBB disruption correlates with the degree of hypoperfusion and is associated with poor collateral flow [102], whereas a favourable penumbral profile that predicts a favourable clinical outcome is associated with reduced BBB disruption [108]. As the clinical stroke severity influences the PSE risk, this suggests a link between BBB disruption and PSE. The findings of a recent study suggested that focal BBB leakage observed in acute ischaemic stroke may be transient and fully reversible after reperfusion [109]. Interestingly, using the DSC-PWI technique for BBB permeability imaging, Arba et al. showed significant BBB leakage in brain areas distal from the ischaemic lesion in acute ischaemic stroke patients with cerebral small vessel disease [110] and this suggests more wide-spread damaging that may be correlated with the generation of PSS. Alternative approaches for assessing BBB disruption in acute stroke include the measurement of the quantitative transverse relaxation time, T2, and the quantitative longitudinal relaxation time, T1. Quantitative T2 is generally sensitive to net water uptake and responds to the increased intracellular and interstitial fluid contents associated with acute stroke [111–114], whereas quantitative T1 is commonly regarded as a sensitive imaging marker for early and subtle BBB disruptions [115]. Previously, both techniques have been successfully implemented in acute stroke patients with reasonable acquisition times for clinical use [111–115]. Consequently, these MRI techniques might be promising candidates for the investigation of PSS and PSE and their associations with BBB leakage, potentially providing deeper pathophysiological insights that contribute to the identification of prevention and treatment strategies.

## Conclusions

The complexity of stroke variables can complicate meaningful clinical trials. However, hypothesis-driven basic studies can pave the way for further research, and prospective, multicentre clinical studies examining large patient collectives with sufficiently standardised detailed information and case numbers to allow for subanalyses are also important. Methodologically sound randomised-controlled trials remain necessary to assess the importance of findings from both basic and observational research in the future.

The current state of PSE research can be summarised as follows:

1. More sophisticated epileptological questions are becoming increasingly important with improved stroke treatment and medical care, requiring detailed scientific investigations to better understand the risk factors associated with epileptogenesis.
2. Currently, no indication exists for the primary prevention of PSS using ASM. Indications for secondary PSS prevention using ASM should be individually determined based on patient characteristics and research findings. Unprovoked LS carry a recurrence risk resulting in PSE diagnosis, and ASM therapy should be offered in these cases. ASM with low pharmacological interaction potential should be preferred.
3. Therapy with rt-PA was initially contraindicated in cases of ASS and later reduced to a relative contraindication. Under individual consideration, ASS does not represent a contraindication to thrombolysis. Current clinical research in epileptology can thus inform the guidelines for stroke treatment.
4. New technologies, such as novel imaging and blood-based biomarkers, may be suitable for assessing PSE risk and therapy effectiveness and open perspectives for further treatment optimisation in an expanding research area of high clinical relevance.

## Data Availability

Relevant data are publicly available from the literature sources. Data aggregated by the authors of this study is available upon reasonable request.

## Abbreviations

AHA: American Heart Association
AMPA: α-Amino-3-hydroxy-5-methyl-4-isoxazolepropionic acid
APTW: Amide proton transfer weighted imaging
ASA: American Stroke Association
ASM: Anti-seizure medication
ASS: Acute symptomatic seizure
AT1: Angiotensin-type 1 receptor
BBB: Blood–brain barrier
CBZ: Carbamazepine
CCA–IMT: Intima-media thickness of the common carotid artery
CEST: Chemical exchange saturation transfer
CRP: C-reactive protein
DAMP: Danger-associated molecular pattern protein
DCE–PWI: Dynamic contrast-enhanced perfusion-weighted magnetic resonance imaging
DOAC: Direct-acting oral anticoagulant
DSC–PWI: Dynamic susceptibility contrast perfusion-weighted magnetic resonance imaging
DWI: Diffusion-weighted magnetic resonance imaging
ECG: Electrocardiogram
EHRA: European Heart Rhythm Association
EPI: Echoplanar imaging
ESL: Eslicarbazepine
ESO: European Stroke Organization
FDA: United States Food and Drug Administration
GBP: Gabapentin
IAT: Intraarterial thrombolysis
ILAE: International League Against Epilepsy
LCM: Lacosamide
LEV: Levetiracetam
LTG: Lamotrigine
LS: Late seizure
MRI: Magnetic resonance imaging
mRS: Modified Rankin Scale
MT: Mechanical thrombectomy
NCAM: Neural cell adhesion molecule
NCSE: Non-convulsive status epilepticus
NIHSS: National Institutes of Health Stroke Scale
OXC: Oxcarbazepine
PER: Perampanel
P-gp: P-glycoprotein
PHT: Phenytoin
PoSERS: Post-Stroke Epilepsy Risk Scale
PSE: Post-stroke epilepsy
PSS: Post-stroke seizure
PWI: Perfusion-weighted magnetic resonance imaging
rt-PA: Recombinant tissue plasminogen activator
SE: Status epilepticus
TGFβ: Transforming growth factor-beta
TNF-R1: Tumour necrosis factor receptor 1
VPA: Valproate
ZNS: Zonisamide
